# Does the EU’s Action Plan for a Circular Economy Challenge the Linear Economy?

**DOI:** 10.1021/acs.est.1c06194

**Published:** 2021-10-29

**Authors:** Nils Johansson

**Affiliations:** KTH Royal Institute of Technology, Department of Sustainable Development, Environmental Science and Engineering, 100 44 Stockholm, Sweden

**Keywords:** circular economy, policy conflict, mining, sustainability transition, European Union

The conventional way of dealing with resources through extraction, production, consumption, and dumping them in landfills are growing into a serious threat to humans and the environment. More than 90% of global biodiversity losses and water stress, 50% of climate emissions, and one-third of the health effects of particulates can be attributed to the extraction and processing of natural resources.^1,2^ To reduce the burden of the conventional, linear way of dealing with resources; decision-makers, companies, NGOs, and the public join forces for a resource transition where natural resources are no longer extracted from and lost to nature, but retained in the economy for as long as possible through circulation.

In March 2020, the EU^1^ launched, as an integrated part of the Green deal, the latest action plan to accelerate the transition to a circular economy. The ambitions are high. EU^1^ shall “lead the way to a circular economy at the global level” (p 3), “reduce its consumption footprint and double its circular material use rate” (p 2). The overall objective is to establish a “regenerative growth model that gives back to the planet more than it takes” (p 2).

To reach these objectives, the action plan^1^ present over 150 measures. Many of these measures demonstrates that policy makers have started to understand and addresses some of the critical challenges of the circular economy. The focus is no longer on recycling but on design, reuse, sharing, durability, and repairing. For example, through ensuring easy access to reuse and repair services (p 10), requirements on design for circulation (p 4) and phasing out single use plastics (p 9). Through standardization (p 14), reducing the complexity of products (p 8), product passports (p 17), and ban on exports (p 14), the focus has also shifted from the actors that generate waste to establishing a functioning market for the customers who shall receive and use the secondary resources and products.

The consumer perspective has also been emphasized by, for example, linking circular economy to social rights (p 15), requirements on product lifetime (p 7) and the right to repair (p 5). The European Commission has also proposed several measures to estimate, control, and monitor the circular economy. For example, by developing harmonized methods for measuring circularity (p 16), minimum requirements for sustainability labels (p 16), conduct inspections (p 5), and counteract green washing (p 5).

However, objectives and measures to shrink the linear economy are absent. The plan contains only marginal resections in the end of the linear economy, for example, restricting only very specific single-use plastics. As a result, the sustainability problems related to waste, in the form of losses of resources and emissions, can be addressed. But, the main sustainability problems related to the linear economy, found at the extraction and processing of virgin materials,^1,2^ remains politically unaddressed.

In fact, in other action plans,^3,4^ the EU states that natural resource extraction shall increase by^3^ providing investments (p 8), develop research programs (p 14), increase the public acceptance (p 7), and facilitate permission processes (p 14). The ambition to increase extraction is primarily driven by geopolitical concerns regarding critical raw materials.

Given the political ambitions to increase recycling, reuse, and sharing, but at the same time also increase extraction, the outflow from the economy may decrease, while the inflow increases. The circular economy may thus not transform the economy from something, only to something new, and develop on top of the extraction economy.

As a result, the circular economy will remain a strategy with remarkably broad political support, which can be implemented without major protests. However, a resource policy with the ambition to simultaneously increase circulation^1^ and natural resource^3,4^ extraction means that the environmental impact in the form of, for example, climate emissions will increase from both the circular economy and the extractive economy. Thereby, the implementation of a circular economy may hinder the fulfillment of the Paris Agreement.

Meeting these two policy targets simultaneously generates also practical ambiguities. For example, should companies’ trials with leasing departments for products be scaled up or freeze as limited ancillary activities to their core business,^5^ based on inexpensive natural resources? Furthermore, the political ambiguity may increase the polarization of natural resource issues. Therefore, decision makers need to make up their mind; shall natural resource extraction increase or decrease? A real transition from a linear to a circular economy is, however, challenging for several reasons:

- The deposits of resources in-use are larger per capita in rich countries, which means that the material conditions for a circular economy varies.
- Natural resource extraction is the backbone of many countries’ and rural local economies, where a decline may have serve social effects.
- Increased global prosperity and a growing middle class will increase the demand for natural resources, which circulation cannot even in theory meet.
- The mining sector has successfully framed their activities as crucial for reaching climate and sustainability goals. For example, increased mining of metals such as lithium is required to meet the demand for renewable energy and digitalization.
- Natural resource extraction is characterized by lock-ins. For example, subsidies make resources in the Earth’s crust more profitable and accessible to extract than the corresponding resources in the built environment.^6^

At the same time, there are several opportunities to trigger a real resource transition:

- Learn from how fossil fuels are phased out by building support, erasing fossil subsidies, formulating radical targets, introducing climate taxes and emission limits, while compensating those affected.
- For a start, formulate quantitative targets for circulation in relation to natural resource extraction. For example, the circular economy strategy of Finland^7^ states: “the total domestic consumption of primary raw materials in 2035 will not exceed the level in 2015”.
- Develop green policy instruments in line with the targets that penalize resource extraction from nature and encourages circulation, for example, through climate taxes and compensation for evaded climate gases.
- Develop tools and models that help decision-makers to navigate and make complex trade-offs between conflicting sustainability ambitions. For example, between the need of extracting metallic minerals to reduce the demand of fossil fuels.
- However, the availability of resources in a circular economy is partly elastic. Above the Earth’s crust, there are significant inactive stocks of resources in, for example, abandoned infrastructure, tailings, landfills and drawers that can meet the increased demands. Hence, the built environment should be prospected before opening new mines.
